# Chronic stridor in a toddler after ingestion of a discharged button battery: a case report

**DOI:** 10.1186/s12887-024-04730-1

**Published:** 2024-04-06

**Authors:** Zoe S. Oftring, Doortje M. Mehrtens, Julian Mollin, Eckard Hamelmann, Sebastian Gaus

**Affiliations:** 1 Department of Paediatrics, University Clinic Giessen & Marburg, Marburg, Germany; 2 Philipps University Marburg and University Clinic Giessen & Marburg, Institute of Digital Medicine, Marburg, Germany; 3 Department of Paediatrics, Bielefeld University, University Hospital OWL, Children’s Center Bethel, Bielefeld, Germany; 4 Clinic Westbrandenburg GmbH, Children’s Hospital, Potsdam, Germany; 5 Pediatric Emergency Department, Bielefeld University, University Hospital OWL, Children’s Center Bethel, Bielefeld, Germany; 6 Notaufnahme Kinderzentrum Bethel (NoKi), Evangelisches Klinikum Bethel (EvKB), Universitätsklinik für Kinder-und Jugendmedizin, Grenzweg 10, Bielefeld, 33617 Germany

**Keywords:** Otolaryngology, Pediatrics, Foreign body, Button battery, Case report

## Abstract

**Background:**

Button battery (BB) ingestions (BBI) are increasingly prevalent in children and constitute a significant, potentially life-threatening health hazard, and thus a pediatric emergency. Ingested BBs are usually charged and can cause severe symptom within 2 h. Discharged BBs ingestion is very rare and protracted symptom trajectories complicate diagnosis. Timely imaging is all the more important. Discharged BBs pose specific hazards, such as impaction, and necessitate additional interventions.

**Case presentation:**

We present the case of a previously healthy 19-month-old girl who was admitted to our pediatric university clinic in Germany for assessment of a three-month history of intermittent, mainly inspiratory stridor, snoring and feeding problems (swallowing, crying at the sight of food). The child’s physical examination and vital signs were normal. Common infectious causes, such as bronchitis, were ruled out by normal lab results including normal infection parameters, negative serology for common respiratory viruses, and normal blood gas analysis, the absence of fever or pathological auscultation findings. The patient’s history contained no evidence of an ingestion or aspiration event, no other red flags (e.g., traveling, contact to TBC). Considering this and with bronchoscopy being the gold standard for foreign body (FB) detection, an x-ray was initially deferred. A diagnostic bronchoscopy, performed to check for airway pathologies, revealed normal mucosal and anatomic findings, but a non-pulsatile bulge in the trachea. Subsequent esophagoscopy showed an undefined FB, lodged in the upper third of the otherwise intact esophagus. The FB was identified as a BB by a chest X-ray. Retrieval of the battery proved extremely difficult due to its wedged position and prolonged ingestion and required a two-stage procedure with consultation of Ear Nose Throat colleagues. Recurring stenosis and regurgitation required one-time esophageal bougienage during follow-up examinations. Since then, the child has been asymptomatic in the biannual endoscopic controls and is thriving satisfactorily.

**Conclusion:**

This case describes the rare and unusual case of a long-term ingested, discharged BB. It underscores the need for heightened vigilance among healthcare providers regarding the potential hazards posed by discharged BBIs in otherwise healthy children with newly, unexplained stridor and feeding problems. This case emphasizes the critical role of early diagnostic imaging and interdisciplinary interventions in ensuring timely management and preventing long-term complications associated even to discharged BBs.

## Background

Button battery (BB) ingestions (BBI) are increasingly prevalent in children [1, 2]. This constitutes a significant, potentially life-threatening health hazard as the electrical current from ingested charged BBs can result in severe burns to the surrounding tissue. This may lead to complications such as vocal cord injuries, aero-digestive mucosal lesions, mediastinitis, strictures, perforations, pneumothoraces, spondylodiscitis, trachea-esophageal or aorto-esophageal fistulas, and life-threatening hemorrhages. 3 V Lithium BBs with a 20 mm diameter account for the majority of severe or fatal outcomes [3–5]. Consequently, BBI constitutes a pediatric emergency [1, 3, 4, 6] and requires urgent removal. Mucosal injury can occur two hours after ingestion, and perforation around twelve hours [3, 4, 6]. Ingested foreign bodies (FB) commonly lodge in the esophagus, especially at the thoracic inlet, aortic arch region, and gastroesophageal junction. This causes symptoms such as retching, vomiting, dysphagia, salivation, and regurgitation. In undetected ingestions, symptoms may be nonspecific, e.g. restlessness, fever, and failure to thrive [1, 7]. This particularly concerns younger children, often leading to delayed or false diagnosis [1, 5].

Ingested BBs are usually charged, making a fast manifestation likely due to the severe, rapid symptom onset. This case report focusses on the rare event of ingestion of discharged BBs. Since discharged BBIs are extremely rare, they do not match the usual diagnostic algorithms routinely used by physicians. This report highlights the diagnostic challenges, namely unspecific, protracted symptom trajectories, and risk of impaction over time and illustrates the hazards associated even with discharged BBs. It concludes that if detection and removal is delayed, additional interventions may be necessary to address complications, such as surgical procedures or repeated esophagoscopic dilatation to treat strictures. Our case report aims to emphasize the need for heightened suspicion and vigilance of discharged BB in children with unexplained chronic stridor as well as early consideration of diagnostic imaging and interdisciplinary management.

## Case presentation

We present the case of a 19-month-old girl who was assigned to our clinic for further investigation of unspecific pulmonary symptoms, stridor and poor feeding – quickly unfolding as the unusual case of a 3-months-old ingestion of a discharged, now impacted BB.

The child was referred to our pediatric gastroenterology clinic by her resident pediatrician for further symptom assessment. The patient’s history had started three months earlier with symptoms of a mild respiratory infection (Table 1). Fever, cough, and rhinitis occurred occasionally thereafter. Several weeks later, mild problems developed concerning swallowing and an inspiratory stridor when crying. Food intake, especially solids, was also reduced during infection-free intervals. Crying attacks occurred when eating or even seeing food.

**Table 1:** Timeline of symptoms and clinical course

Month	Symptoms / Progress	Management / Findings
0	Symptom onset with coughing, rhinitis similar to a mild respiratory infection Subsequently, occasionally recurring fever, cough, and rhinitis	/
+ 1 month	Development of mild feeding problems (impaired swallowing, crying when eating) as well as development of an inspiratory stridor	/
	Presentation of stridor during routine check-up with resident pediatric	- Prescription of inhalation therapy with Salbutamol - Referral to ENT for further assessment
+ 2 months	Appointment at ENT Stable condition, symptoms unchanged	- Visualization of upper laryngo-pharynx without any suspicious findings - Inability to visualize epiglottis level - Referral to ENT clinic
	Appointment at ENT hospital department Stable condition, symptoms unchanged	- Endoscopic examination of airway tract up to the epiglottis without any suspicious findings - Recommendation to continue therapy with inhalatives and corticosteroid suppositories - Recommendation for bronchoscopy
+ 3 months	Inpatient stay at our pediatric university clinic for further assessment Stable condition, symptoms unchanged	- Diagnostic bronchoscopy reveals airway obstruction, subsequent esophagoscopy shows unidentifiable FB - Chest X-ray identifies FB as button battery - First endoscopic salvage attempt, unsuccessful due to complexly lodged position - Second endoscopic salvage attempt with ENT colleagues and rigid esophagoscope, successful removal
+ 5 months	Follow-up esophagoscopy in our clinic Child in good condition but with occasional swallowing difficulties	Visualization of moderate stenosis Bougienage due to feeding difficulties
+ 6,5 months & + 8 months	Follow-up esophagoscopy in our clinic Child in good, asymptomatic condition, thriving	- Visualization of moderate stenosis - No bougienage due to asymptomatic child - biannual controls since

One month after symptom onset, the child visited her resident pediatrician for a routine check-up. The doctor observed the stridor and prescribed inhalation therapy with Salbutamol. Additionally, he referred the child to an outpatient ear nose and throat (ENT) specialist. The ENT examination, a few weeks later, showed no nasopharyngeal or oral abnormalities. However, the larynx could not be examined, and the ENT referred the child to an ENT hospital clinic for further assessment. There, an endoscopy up to the epiglottis revealed no suspicious findings. Continuation of inhalation therapy with sympathomimetics and cortisone suppositories as well as a diagnostic bronchoscopy was recommended.

For this, the girl was taken to our clinic. She presented mildly agitated, in good health, with a weight of 9.75 kg (15. percentile), and height of 79.6 cm (15. percentile). Physical examination revealed normal lung auscultation with bilaterally equal vesicular breathing sounds, inspiratory stridor when crying, and otherwise normal findings. SpO2 was 98% and body temperature 37.4 °C, remaining vital signs were normal. Further anamnesis revealed no chronic infections or diseases, no allergies, no previous surgeries, or intubations, no known genetic disorders. Her motor, psycho-social and speech development was age-appropriate. Labs (blood count, liver, kidney, inflammation parameters, coagulation) showed mild leucocytosis, anemia, and thrombocytosis, but negative infection markers. She snored whilst sleeping, but SpO2 levels remained stable.

The child’s medical history was empty for aspiration or ingestion of FB or dangerous liquids, or other red flags. Given the child’s age, laryngo- or tracheomalacia was unlikely. Other differential diagnoses included newly developed stenosis, e.g., due to hemangioma, or neoplasm, which warranted a diagnostic bronchoscopy. In the pre-existing records from the outpatient setting, there was no indication that imaging (X-ray) was considered, and there was a greater expectation of diagnostic value performing an elective bronchoscopy to clarify the patient's symptoms.

**Fig. 1:**
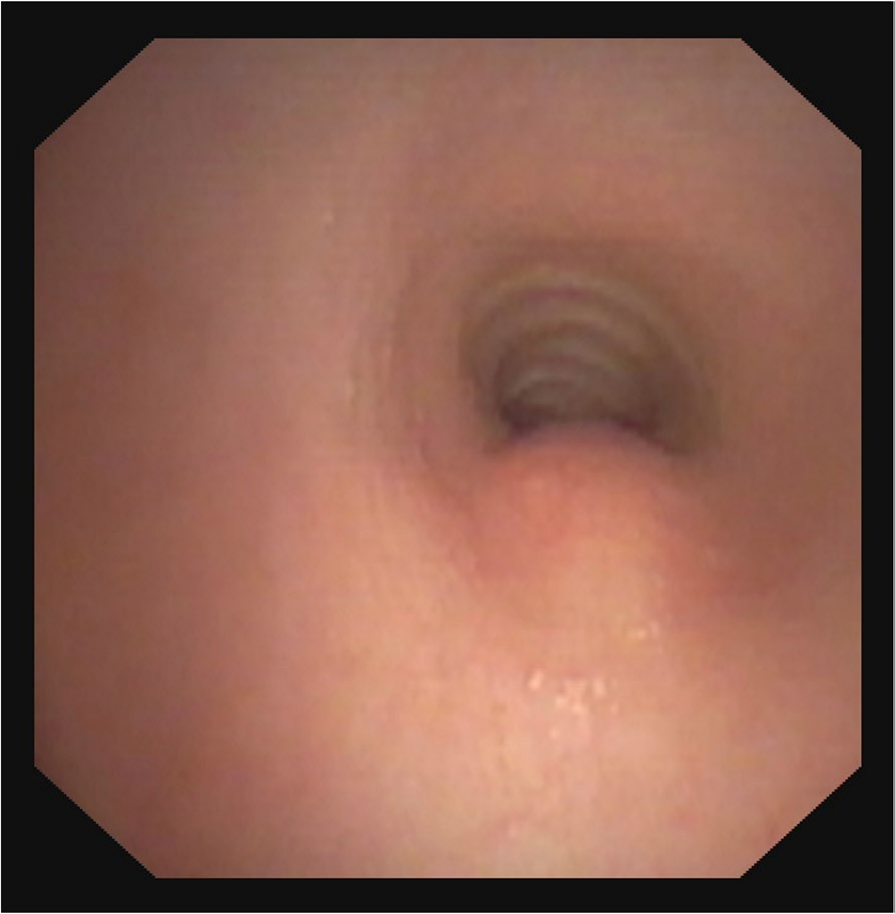
Bronchoscopic image of the trachea showing a non-pulsative bulge protruding into the lumen and otherwise normal mucosal structures

The intervention was conducted on the pediatric intensive care unit (PICU) in the PICU’s dedicated endoscopy room by a multiprofessional team consisting of specialists from pediatric and adult endoscopy, pediatric intensive care, pediatric surgery, and pediatric anesthesia. All team members had extensive training and experience in the procedure. Bronchoscopy showed an overall normal bronchial structure except for a non-pulsatile stenosis in the middle of the trachea (Fig. 1), prompting a diagnostic esophagoscopy directly afterwards.

**Fig. 2:**
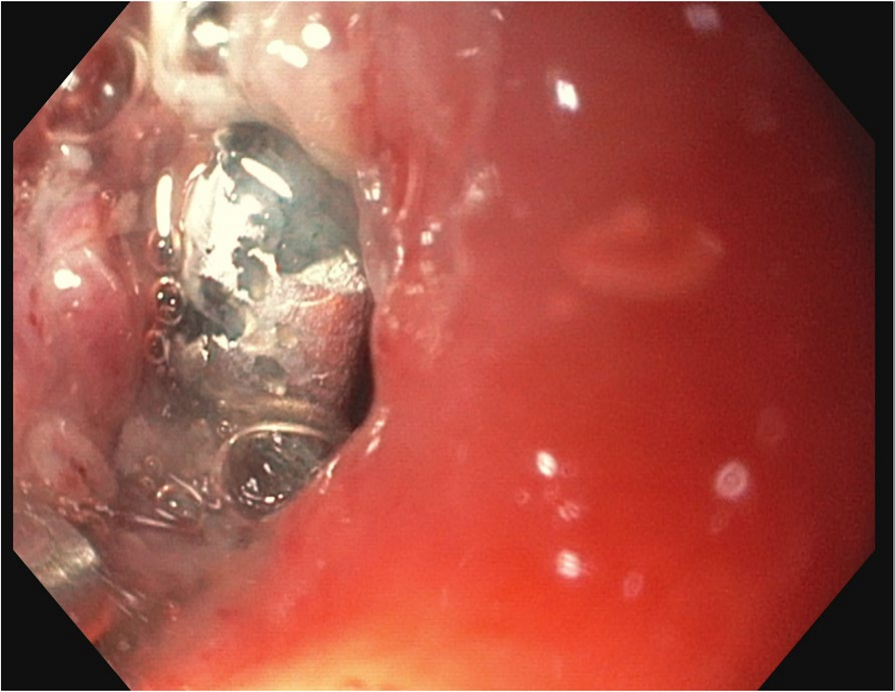
Endoscopic image of the upper esophagus showing an unidentifiably foreign body, corresponding to the impression on the trachea, which obstructs the esophageal lumen

**Fig. 3:**
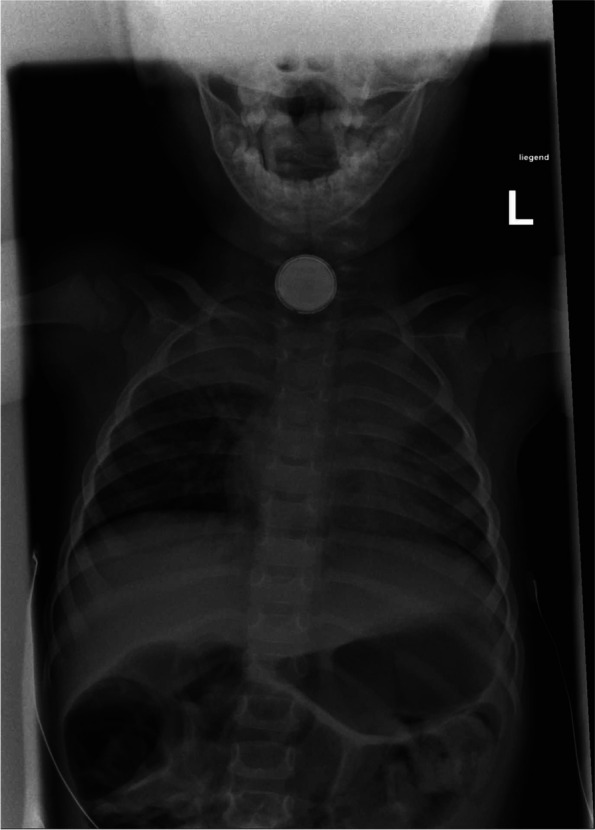
Chest x-ray in AP view of button battery in the upper mediastinum, showing the typical “halo” or “double ring” sign with two concentric circles. The BB is projecting onto the upper part of the esophagus. The narrow end depicts the negative pole and is facing ventrally

This revealed an undefined, metallic FB lodged in the upper third of the esophagus (Fig. 2). The FB resembled a BB, however, due to the long impaction time, this seemed unlikely. For clarification, we conducted a chest X-ray. This identified a BB above the proximal esophageal junction (Fig. 3). To secure the airway, the child was then nasally intubated.

Directly afterwards, we attempted a flexible endoscopic retrieval, using grasping forceps, snares and traps. As this proved unsuccessful, we switched to rigid bronchoscopy, laryngoscope and Magill forceps. Anatomic structures further complicated wedging and made salvage extremely difficult. Due to increasing edematous swelling of the area, further attempts were terminated after several hours, and the intubated and sedated child remained in a patient room at our PICU. On the same day, the ENT department was consulted to perform a rigid esophagoscopy the next day after swelling declined.

The following day, the ENT team (consisting only of consultants with year-long experience) conducted the procedure in theater. For this, the child was put under general anesthesia. The battery was fixated with large grasping forceps. Extraction remained challenging, required considerable force, but eventually proved successful. After retrieval of a type 2032 BB, a gastric tube was left in place to splint the esophagus. The patient remained stable at all times and was transferred back to our PICU for ventilation and monitoring. Post-operative care included intravenous antibiotics to prevent mediastinitis (Cefuroxime 150 mg/kgKG bw/d, Metronidazole 30 mg/kg bw/d, Gentamicin 5 mg/kgKG bw/d), fresh frozen plasma due to mildly abnormal coagulation parameters and regarding the extensive esophageal manipulation, and enteral feeding through a gastric tube to allow the esophageal mucosa to heal.

Five days post intervention, an esophageal fluoroscopy revealed no signs of perforation, and the patient was extubated. Antibiotic prophylaxis was administered for two more days (in total 7 days). Inflammation markers (CRP) peaked at 44.8 mg/l on the day of FB extraction and decreased rapidly. Seven days after removal, a stepwise oral reinduction of liquids was commenced. Eleven days post intervention, a control endoscopy showed a circumscribed, fibrin-covered incipient scarring erosion, but no evidence of ulceration, pocket formation, or stenosis. Solids were gradually reintroduced and eating and drinking behavior remained complication-free. After feeding tube removal on day 14, the girl was discharged the following day.

A detailed review of the disease trajectory and reevaluation with the mother revealed that an unobserved sudden coughing attack without cyanosis had occurred in the context of a respiratory infection three months prior to presenting to our clinic. It is possible that this constituted the unobserved BBI.

### Follow-up care

**Fig. 4:**
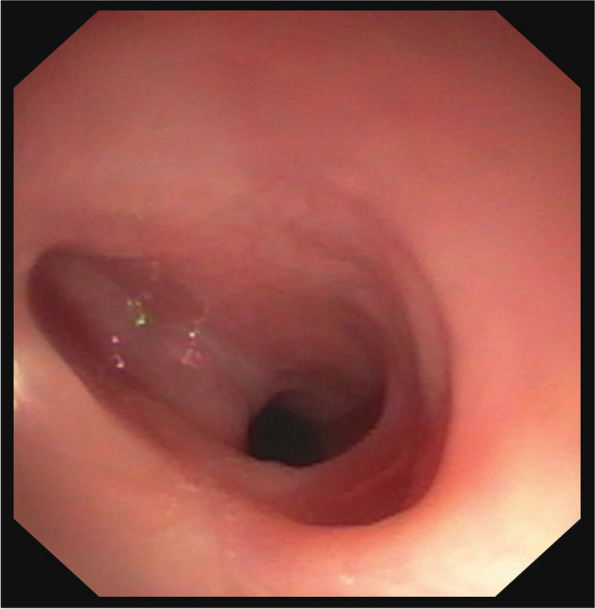
Endoscopic follow up control. Image of the upper esophagus mucosa (10 cm from the dental arch) showing the persisting pocket (on the left in the picture)

Two months later, a follow-up revealed no stridor, but regurgitation happened occasionally, especially with solids. Esophagoscopy showed a narrowing of 10 mm in diameter at 10 cm from the dental arch, and a lateral pocket formation above (Fig. 4), prompting bougienage with dilators up to 13 mm. Afterwards, gradual reintroduction of food was complication-free. Another control endoscopy 6 weeks later showed a 10 mm stenosis and a persistent pocket. Since the child had been asymptomatic, no bougienage was performed this time. The following control endoscopy another 6 weeks later revealed a persistent but short-segment stenosis. The pocket remained unchanged. It is assumed that the pocket will stretch with growth. The latest follow-up examinations also point in this direction. Unlike larger pockets, food residue accumulation is not a concern in this instance. Currently, endoscopic controls are performed biannually as the patient remains asymptomatic and is thriving according to age. No bougienage has been performed since. If the situation remains stable, the monitoring intervals will be extended. Overall, the follow-up has been and continues to be uncomplicated, challenges occurred at no time and the family and patient were compliant and attended the appointments reliably at all times.

### Patient perspective

The parents reported a positive relationship with the healthcare team, characterized by effective and transparent communication at eye level at all times both during the acute treatment of the BBI as well as during the follow up visits. They expressed satisfaction with the empathetic, collaborative and constructive approach aimed at the child's welfare.

## Discussion

FB ingestions constitutes a common problem in pediatrics, with BBIs making up 5%–11% [8–11] of all FB ingestions. Toddlers ≤ 6 years [1, 3, 12, 13] are at the highest risk for BBIs, accounting for 57–80% of cases, incidence rates in older children are lower [12–14]. BBIs have complication rates between 0.166%–12.6% [3, 15], and a lethality of 0.04% [15]. Due to initially vague symptoms, diagnosis is often delayed [3, 16]. In our case, in hindsight, all the child's symptoms can be attributed to the esophageal BB obstruction. Retrospectively, the nocturnal breathing noises described as snoring – normally caused by upper airway obstruction – are more likely to be due to an obstruction of the lower airways caused by the protruding BB in the esophagus. Nevertheless, initially, BBI seemed very unlikely due to the atypical, prolonged trajectory and anamnesis unindicative of FB. Unspecific symptoms and newly developed stridor justified a diagnostic bronchoscopy, especially as bronchoscopy constitutes the most sensitive and accurate method for diagnosing FBs [17, 18]. Radiographs  do not often offer additional insight for acute stridor [19], and its benefit is equivocal, especially in radiolucent FBs [18, 20]. However, in chronic cases like this, a prior chest x-ray would have been warranted [20, 21].

It remains unclear to what extent anatomical features of the lodged position impeded recovery. The proximal portion of the esophagus, where the BB was lodged, is built from striated muscle. This together with the complex neuronal innervation in this region may have influenced the clinical outcome and the difficult extraction of the BB. Since endoscopy showed no signs of ulceration in our case, mucosal destruction, or fistulation, the BB must have been discharged upon ingestion. While BBIs are common, prolonged courses without detrimental consequences are very rare. To the best of our knowledge, this is the first case of refractory chronic stridor due to unwitnessed, prolonged BBI. Two reports describe cases of toddlers with a 3-months history of swallowing difficulties after unwitnessed BBI [22, 23]. Contrary to our case, they developed no consequences such as strictures after removal. Alam et al. report the case of a 2-year-old child with swallowing difficulties over 7 months, eventually diagnosed as BBI. The boy survived but required thoracotomy for removal [24]. Several publications of prolonged BBI report fatalities due to trachea-esophageal fistulation or sudden hemorrhage after vasculo-esophageal fistulation [3, 25–28].

Relevant points can be learnt: Firstly, in children < 6 years with prolonged respiratory symptoms or feeding problems, FB ingestion or aspiration needs to be considered. Secondly, while extended asymptomatic BBI is rare, its consideration is important given the prevalence of BBI in toddlers. Aligning with this, in cases of unclear, chronic stridor, a chest x-ray is warranted. Furthermore, in cases of ambiguous symptoms, or new respiratory/feeding issues, a thorough anamnesis is essential. This should include asking about (i) the presence of BBs at home, and (ii) signs of FB aspiration/ingestion. Thirdly, in complex FB infestation, early consultation with ENT specialists is recommended for their expertise with additional instruments like rigid esophagoscopy. This should occur across hospital boundaries, as children may need to be transferred or specialized equipment brought into the clinics. Lastly, but nevertheless urgently, the frequency and severity of BBIs warrant greater industry efforts to mitigate risks. Preventive approaches already exist (e.g., safer packaging and battery design, special coating) [2, 7, 29]. However, prevention is always better than treatment, especially in cases of foreign body ingestion in a vulnerable group such as toddlers. Thus, stricter product standards, legal [30] and governmental regulations and heightened public awareness are strongly recommended.

## Conclusion

Unwitnessed BBIs in children presents a diagnostic challenge. Although life-threatening hazards such as ulceration and perforation are unlikely in discharged BBs, they still pose a significant risk, and may lead to long-term complications as observed here. BBI should be considered in children with newly developed airway and feeding problems. An early chest x-ray is warranted in pediatric stridor of unknown origin. Children with prolonged BBI are at risk of a complexly lodged FB, and highly benefit from an interdisciplinary endoscopic salvage approach.

## Data Availability

All relevant data is included in the case report. Further information are available from the corresponding author on reasonable request.

## Abbreviations

BB: Button battery
BBI: Button battery ingestion
ENT: Ear, nose and throat
FB: Foreign body
PICU: Pediatric intensive care unit
